# Molecular Phylogeny of the Butterfly Genus *Polytremis* (Hesperiidae, Hesperiinae, Baorini) in China

**DOI:** 10.1371/journal.pone.0084098

**Published:** 2013-12-31

**Authors:** Weibin Jiang, Jianqing Zhu, Chao Song, Xiaoyan Li, Yue Yang, Weidong Yu

**Affiliations:** 1 Shanghai Normal University, College of Life and Environmental Sciences, Shanghai, People’s Republic of China; 2 Shanghai Zoological Park, Shanghai, People’s Republic of China; University of Innsbruck, Austria

## Abstract

**Background:**

The genus *Polytremis*, restricted to the continental part of the southeastern Palaearctic and northern Oriental regions, is one of the largest and most diverse lineages of the tribe Baorini. Previous studies on the genus were focused mainly on morphological classification and identification of new species. Due to the lack of effective and homologous traits of morphology, there were many challenges in the traditional classification. In this report, we reconstruct the phylogeny to provide a solid framework for these studies and to test the traditional limits and relationships of taxa.

**Methodology and Principal Findings:**

We sequenced a mitochondrial and three nuclear gene fragments, coupled with an evaluation of traditional morphological characters, to determine the phylogenetic relationships for a total of 15 species representing all major species groups of the *Polytremis* genus in China, and to elucidate their taxonomic status.

**Conclusions and Significance:**

Analysis of mitochrondial and nuclear DNA showed considerable congruent phylogenetic signal in topology at the inter-species level. We found strong support for the monophyly of *Polytremis* and some clades were recognized with morphological data. Thus, the COI sequence in our study could be used as a DNA barcode to identify almost all members of the genus. However, incongruences of phylogenetic analyses occurred: in contrast to the phylogenetic trees of mitochondrial COI, it was not possible for nuclear rDNA to discriminate *P. gotama* from *P. caerulescens*, suggesting a possible recent separation of these two species. Additionally, *P. theca* was the only species with a greater intra-specific genetic distance compared to some inter-specific genetic distances in this study and some problems associated with the cryptic diversity of the species are discussed. The results of this study will helpful to reveal the causes of the high degree of diversity of butterflies, and possibly other groups of insects in China.

## Introduction

Butterflies have long served as a model system for ecological and evolutionary studies on the basis of the high degree of diversity and complexity [1]. The family Hesperiidae, commonly known as “skippers” includes around 4000 species, of which Hesperiinae is the largest subfamily. Baorini is a tribe of subfamily Hesperiinae and one of the largest and most diverse lineages of the tribe. The genus *Polytremis* Mabille, 1904 has a thick body and relatively small wings. These wings are commonly yellowish brown or dark brown [2]. The main external features are characterized by serial, linear, semi-hyaline spots and the absence of a cell spot on the underside of each hindwing (except *P. gotama* Sugiyama, 1999 which has a silver short line in discal cell), as well as the unspined mesotibia. Male genitalia are distinguished by the swollen tegumen, bifid uncus and elongated harpe [3], [4].

The genus *Polytremis* has 18 members and is restricted geographically to the continental part of the southeastern Palaearctic and northern Oriental regions. These species, except *P. minuta* and *P. annama*, were described in China, including 11 Chinese endemic species [3]–[7]. Evans [5] recognized 12 species and the other 6 species were reported in recent years [2]–[4], [6]. Since a majority of the species is gathered in southern China, we suggest this region could be the primary area where *Polytremis* originated and separated.

Earlier studies of the genus *Polytremis* were focused mainly on morphological classification, population distribution and identification of new species. There is a lack of effective and homologous traits of morphology in most species of the family Hesperiidae. Similarly, there were many challenges in the traditional classification system and phylogenetic relationship based on morphological classification in the genus *Polytremis* [8], [9]. The inherent defects of phenotypic plasticity and genetic variability in traditional classification can also lead to lack of effective identification. [10], [11].

Analysis of DNA has been widely used in the phylogenetic studies of the family Hesperiidae using mitochondrial cytochrome *c* oxidase I (COI) and II (COII) genes or nuclear Wingless, EF-1α genes. [12], [13]. These methods are more effective and specific than traditionally morphological methods in genetic variation and phylogenetic relationships involved in sibling species, cryptic and morphologically intermediate species [14], [15]. In this study, we constructed the molecular phylogenetic trees of mitochondrial gene COI and three parts of nuclear genes termed the D3 region of 28S rDNA and the V4 and V7 regions of 18S rDNA from the Chinese species of *Polytremis*. In addition, we discuss how far the results of molecular phylogeny are congruent with traditional classification based on morphology. We also explore the feasibility of the mitochondrial COI gene as a DNA barcode for classification and identify members of the genus *Polytremis*. Consequently, we tested 67 *Polytremis* spp. specimens by molecular techniques to: (1) discuss the relationships between the genetic lineages and morphological variation; (2) investigate whether DNA barcodes can be used to identify *Polytremis* spp. conveniently and accurately; and (3) assess whether there is cryptic diversity or the existence of morphological intermediates in the genus *Polytremis.*

## Results

**Figure 1:**
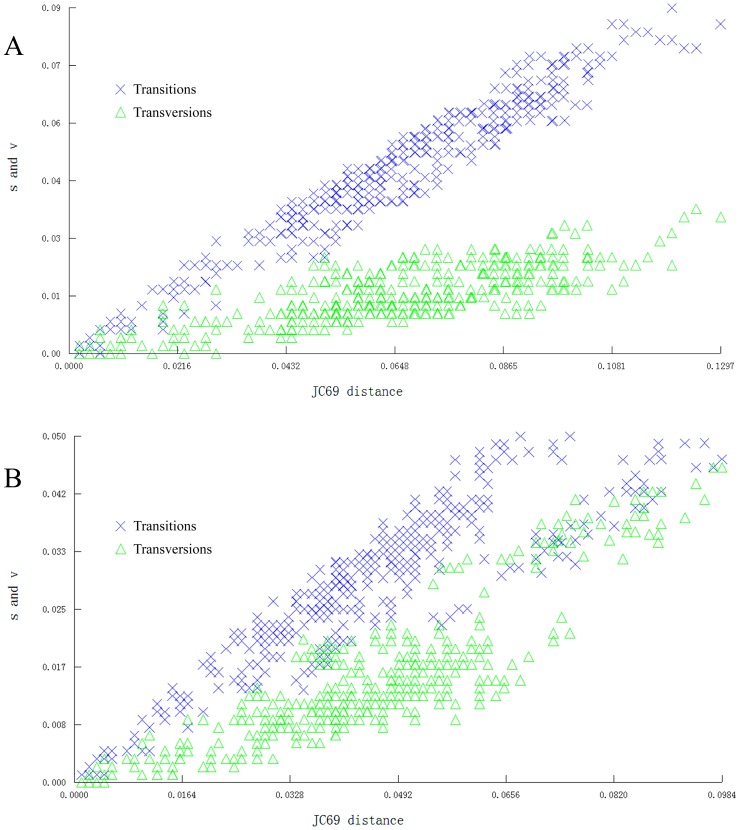
Transitions and transversions plotted against Kimura-2-parameter distance. (A) the mtDNA cytochrome *c* oxidase I (COI) fragment in *Polytremis*; (B) the concatenated sequences (mt DNA COI+nuclear rDNA).

COI and rDNA (D3+V4+V7) from all specimens were amplified and sequenced. The results of the nucleotide substitution model detected with software DAMBE (Data Analysis in Molecular Biology & Evolution) show that the COI and the concatenated sequences (COI+rDNA) do not exhibit saturation in either transition or transversion and can be used for further phylogenetic analysis (Fig. 1).

### (a) Genetic Divergences

The data set of COI alignment contains 490 nucleotide positions, of which 144 positions are variable and 97 are parsimony informative. The mean base composition of the fragment shows a strong bias of A+T (T 39.3%, C 18.5%, A 28.6% and G 13.6%), as found commonly in insect mitochondrial genomes [16]. In all, 31 haplotypes of *Polytremis* and outgroups were found and deposited in GenBank with accession numbers KC684389–KC684419 (Table 1). The average inter-specific K2P distance is 7.9% and the shortest distance was observed between *P. gigantea* Tsukiyama, 1997 and *P. suprema* Sugiyama, 1999 (K2P distance 1.7%). The intra-specific distance ranges up to 4.2% for the *P. theca* Evans, 1937. The specimens of their subspecies *P. theca theca* Evans, 1937 and *P. theca fukia* Evans, 1940 are characterized by K2P distances ranging up to 1.9% and 2.6%, respectively.

**Table 1:** List of taxa and voucher specimens used for sequencing.

Species	Sample no.	Sex	Locality (Code in Fig.7)	Coordinates	Date	Haplotype(number in network)	Accession no. COI	Accession no. D3/V4/V7 (rDNA)
*Polytremis nascens*	121107001	male	Baoxing, Sichuan (BX)	30°22′N 102°47′E	5-Jul-09	*P. nascens* A (9)	KC684397	KC684338/KC684355/KC684372
*Polytremis nascens*	121107002	male	Tianquan, Sichuan (TQ)	30°03′N 102°45′E	3-Sep-10	*P. nascens* A (9)	KC684397	KC684338/KC684355/KC684372
*Polytremis nascens*	121107003	female	Tianquan, Sichuan (TQ)	30°03′N 102°45′E	3-Sep-10	*P. nascens* A (9)	KC684397	KC684338/KC684355/KC684372
*Polytremis nascens*	121107008	male	Tianquan, Sichuan (TQ)	30°03′N 102°45′E	3-Sep-10	*P. nascens* A (9)	KC684397	KC684338/KC684355/KC684372
*Polytremis nascens*	130101053	male	Tianquan, Sichuan (TQ)	30°03′N 102°45′E	3-Sep-10	*P. nascens* A (9)	KC684397	KC684338/KC684355/KC684372
*Polytremis nascens*	121107004	female	Jinxiu, Guangxi (JX)	27°04′N 110°11′E	26-Jul-11	*P. nascens* B (10)	KC684398	KC684338/KC684355/KC684372
*Polytremis nascens*	121107005	female	Jinxiu, Guangxi (JX)	27°04′N 110°11′E	26-Jul-11	*P. nascens* B (10)	KC684398	KC684338/KC684355/KC684372
*Polytremis nascens*	121107006	male	Lingui, Guangxi (LG)	25°14′N 110°12′E	16-Jul-11	*P. nascens* B (10)	KC684398	KC684338/KC684355/KC684372
*Polytremis nascens*	121107007	female	Suiyang, Guizhou (SY)	27°56′N 107°10′E	15-Aug-10	*P. nascens* B (10)	KC684398	KC684338/KC684355/KC684372
*Polytremis nascens*	130101054	male	Qingyuan, Zhejiang (QY)	27°37′N 119°03′E	21-Jul-07	*P. nascens* B (10)	KC684398	KC684338/KC684355/KC684372
*Polytremis nascens*	130101055	male	Suiyang, Guizhou (SY)	27°56′N 107°10′E	16-Aug-10	*P. nascens* B (10)	KC684398	KC684338/KC684355/KC684372
*Polytremis nascens*	130101056	male	Lingui, Guangxi (LG)	25°14′N 110°12′E	16-Jul-11	*P. nascens* B (10)	KC684398	KC684338/KC684355/KC684372
*Polytremis pellucid*	121112009	female	West Tianmu Mountains, Zhejiang (WTMM)	30°20′N 119°23′E	19-Sep-08	*P. pellucid* A (5)	KC684393	KC684348/KC684365/KC684382
*Polytremis pellucid*	121112013	female	West Tianmu Mountains, Zhejiang (WTMM)	30°20′N 119°23′E	19-Sep-08	*P. pellucid* A (5)	KC684393	KC684348/KC684365/KC684382
*Polytremis pellucid*	121112016	female	West Tianmu Mountains, Zhejiang (WTMM)	30°20′N 119°23′E	19-Sep-08	*P. pellucid* A (5)	KC684393	KC684348/KC684365/KC684382
*Polytremis pellucid*	121112020	female	West Tianmu Mountains, Zhejiang (WTMM)	30°20′N 119°23′E	19-Sep-08	*P. pellucid* B (6)	KC684394	KC684348/KC684365/KC684382
*Polytremis theca fukia*	121112010	male	Wuyi Mountains, Fujian (WY)	27°44′N 118°01′E	19-Sep-08	*P. theca fukia* A (19)	KC684408	KC684342/KC692371/KC684376
*Polytremis theca fukia*	130101057	male	Wuyi Mountains, Fujian (WY)	27°44′N 118°01′E	19-Sep-08	*P. theca fukia* A (19)	KC684408	KC684342/KC692371/KC684376
*Polytremis theca fukia*	121112012	female	West Tianmu Mountains, Zhejiang (WTMM)	30°20′N 119°23′E	8-May-09	*P. theca fukia* B (20)	KC684409	KC684342/KC692371/KC684376
*Polytremis theca fukia*	121112015	male	West Tianmu Mountains, Zhejiang (WTMM)	30°20′N 119°23′E	20-Sep-08	*P. theca fukia* B (20)	KC684409	KC684342/KC692371/KC684376
*Polytremis theca fukia*	130101058	male	West Tianmu Mountains, Zhejiang (WTMM)	30°20′N 119°23′E	17-Aug-09	*P. theca fukia* B (20)	KC684409	KC684342/KC692371/KC684376
*Polytremis theca fukia*	121119045	female	Jingang Mountains, Jiangxi (JGM)	26°45′N 114°17′E	19-Oct-10	*P. theca fukia* C (17)	KC684406	KC684342/KC692371/KC684376
*Polytremis theca fukia*	121112017	female	Jingang Mountains, Jiangxi (JGM)	26°45′N 114°17′E	19-May-10	*P. theca fukia* C (17)	KC684406	KC684342/KC692371/KC684376
*Polytremis theca fukia*	121112019	female	Jingang Mountains, Jiangxi (JGM)	26°45′N 114°17′E	20-May-10	*P. theca fukia* C (17)	KC684406	KC684342/KC692371/KC684376
*Polytremis theca fukia*	130101059	female	Xing’an, Guangxi (XA)	25°37′N 110°40′E	8-Jul-11	*P. theca fukia* D (18)	KC684407	KC684342/KC692371/KC684376
*Polytremis theca theca*	121119046	female	Tianquan, Sichuang (TQ)	30°03′N 102°45′E	6-Jul-11	*P. theca theca* A (21)	KC684410	KC684342/KC684359/KC684376
*Polytremis theca theca*	121119048	male	Tianquan, Sichuang (TQ)	30°03′N 102°45′E	11-Aug-11	*P. theca theca* A (21)	KC684410	KC684342/KC684359/KC684376
*Polytremis theca theca*	121119047	male	Wenchuan, Sichuan (WC)	31°29′N 103°35′E	6-Aug-11	*P. theca theca* B (22)	KC684411	KC684342/KC684359/KC684376
*Polytremis theca theca*	130101060	male	Wenchuan, Sichuan (WC)	31°29′N 103°35′E	6-Aug-11	*P. theca theca* B (22)	KC684411	KC684342/KC684359/KC684376
*Polytremis theca theca*	130101061	male	Luding, Sichuan (LD)	29°55′N 102°15′E	19-Jul-11	*P. theca theca* B (22)	KC684411	KC684342/KC684359/KC684376
*Polytremis theca theca*	121119049	female	Feng County, Shanxi (FC)	33°54′N 106°31′E	19-Jul-11	*P. theca theca* C (24)	KC684413	KC684342/KC684359/KC684376
*Polytremis theca theca*	121119050	male	Feng County, Shanxi (FC)	33°54′N 106°31′E	16-Jul-10	*P. theca theca* C (24)	KC684413	KC684342/KC684359/KC684376
*Polytremis theca theca*	130101062	male	Qinling Mountains, Shanxi (QLM)	34°14′N 103°54′E	16-Jul-10	*P. theca theca* D (23)	KC684412	KC684342/KC684359/KC684376
*Polytremis mencia*	121112011	male	West Tianmu Mountains, Zhejiang (WTMM)	30°20′N 119°23′E	30-May-10	*P. mencia* A (25)	KC684414	KC684347/KC684364/KC684381
*Polytremis mencia*	121112014	male	West Tianmu Mountains, Zhejiang (WTMM)	30°20′N 119°23′E	5-Jun-11	*P. mencia* B (25)	KC684415	KC684347/KC684364/KC684381
*Polytremis mencia*	121112018	male	West Tianmu Mountains, Zhejiang (WTMM)	30°20′N 119°23′E	15-Jun-10	*P. mencia* B (25)	KC684415	KC684347/KC684364/KC684381
*Polytremis mencia*	130101063	female	West Tianmu Mountains, Zhejiang (WTMM)	30°20′N 119°23′E	20-Sep-08	*P. mencia* C (26)	KC684416	KC684347/KC684364/KC684381
*Polytremis mencia*	130101064	male	Ningbo, Zhejiang (NB)	29°52′N 121°32′E	23-Aug-11	*P. mencia* D (27)	KC684417	KC684347/KC684364/KC684381
*Polytremis zina*	121116021	male	West Tianmu Mountains, Zhejiang (WTMM)	30°20′N 119°23′E	18-Aug-09	*P. zina* (7)	KC684395	KC684350/KC684367/KC684384
*Polytremis zina*	121116022	male	Xing’an, Guangxi (XA)	25°37′N 110°40′E	24-Jul-12	*P. zina* (7)	KC684395	KC684350/KC684367/KC684384
*Polytremis zina*	121116023	male	Xing’an, Guangxi (XA)	25°37′N 110°40′E	24-Jul-12	*P. zina* (7)	KC684395	KC684350/KC684367/KC684384
*Polytremis discreta*	121116024	male	Xuanwei, Yunnan (XW)	26°13′N 104°06′E	6-Aug-10	*P. discreta* (4)	KC684392	KC684349/KC684366/KC684383
*Polytremis discreta*	121116025	male	Baoxing, Sichuan (BX)	30°03′N 102°45′E	Jun, 2008	*P. discreta* (4)	KC684392	KC684349/KC684366/KC684383
*Polytremis discreta*	121116026	male	Dulong River, Yunnan (DLR)	27°44′N 98°20′E	5-Jun-09	*P. discreta* (4)	KC684392	KC684349/KC684366/KC684383
*Polytremis lubricans*	121116027	male	Baoxing, Sichuan (BX)	30°22′N 102°47′E	Jun, 2008	*P. lubricans* (3)	KC684391	KC684344/KC684361/KC684378
*Polytremis lubricans*	121116028	female	Xing’an, Guangxi (XA)	25°37′N 110°40′E	6-Jul-11	*P. lubricans* (3)	KC684391	KC684344/KC684361/KC684378
*Polytremis lubricans*	121116029	female	Yuanjiang, Yunnan (YJ)	23°36′N 101°59′E	4-Oct-11	*P. lubricans* (3)	KC684391	KC684344/KC684361/KC684378
*Polytremis eltola*	121116030	male	Jingxiu, Guangxi (JX)	24°07′N 110°11′E	28-Jul-11	*P. eltola* A (1)	KC684389	KC684345/KC684362/KC684379
*Polytremis eltola*	121116031	female	Jingxiu, Guangxi (JX)	24°07′N 110°11′E	21-Jul-11	*P. eltola* A (1)	KC684389	KC684345/KC684362/KC684379
*Polytremis eltola*	121116032	male	Dulong River, Yunnan (DLR)	27°44′N 98°20′E	5-Jun-09	*P. eltola* B (2)	KC684390	KC684345/KC684362/KC684379
*Polytremis gigantea*	121119033	female	Qingcheng Mountains, Sichuan (QCM)	30°53′N 103°34′E	28-Aug-11	*P. gigantea* (14)	KC684403	KC684346/KC684363/KC684380
*Polytremis gigantea*	121119034	male	Nanling, Guangdong (NL)	24°55′N 113°00′E	9-Jul-09	*P. gigantea* (14)	KC684403	KC684346/KC684363/KC684380
*Polytremis gigantea*	121119035	male	West Tianmu Mountains, Zhejiang (WTMM)	30°20′N 119°23′E	18-Aug-06	*P. gigantea* (14)	KC684403	KC684346/KC684363/KC684380
*Polytremis matsuii*	121119036	male	Hongya, Sichuan (HY)	29°54′N 103°22′E	4-Jun-11	*P. matsuii* (12)	KC684400	KC684339/KC684356/KC684373
*Polytremis matsuii*	121119037	female	Hongya, Sichuan (HY)	29°54′N 103°22′E	7-Jul-11	*P. matsuii* (12)	KC684400	KC684339/KC684356/KC684373
*Polytremis matsuii*	121119038	male	Nanling, Guangdong (NL)	24°55′N 113°00′E	23-Jun-07	*P. matsuii* (12)	KC684400	KC684339/KC684356/KC684373
*Polytremis caerulescens*	121119039	male	Tianquan, Sichuan (TQ)	30°03′N 102°45′E	21-Jul-11	*P. caerulescens* (11)	KC684399	KC684352/KC684369/KC684386
*Polytremis caerulescens*	121119040	female	Tianquan, Sichuan (TQ)	30°03′N 102°45′E	6-Jul-11	*P. caerulescens* (11)	KC684399	KC684352/KC684369/KC684386
*Polytremis caerulescens*	121119041	male	Weixi, Yunnan (WX)	27°10′N 99°07′E	9-Jul-10	*P. caerulescens* (11)	KC684399	KC684352/KC684369/KC684386
*Polytremis jigongi*	121119042	male	West Tianmu Mountains, Zhejiang (WTMM)	30°20′N 119°23′E	11-Jul-09	*P. jigongi* A (15)	KC684404	KC684343/KC684360/KC684377
*Polytremis jigongi*	121119043	male	West Tianmu Mountains, Zhejiang (WTMM)	30°20′N 119°23′E	26-Jun-09	*P. jigongi* B (16)	KC684405	KC684343/KC684360/KC684377
*Polytremis jigongi*	121119044	male	Qinliang Peak, Zhejiang (QLP)	30°05′N 118°52′E	9-Aug-11	*P. jigongi* B (16)	KC684405	KC684343/KC684360/KC684377
*Polytremis jigongi*	130101065	male	West Tianmu Mountains, Zhejiang (WTMM)	30°20′N 119°23′E	11-Jul-09	*P. jigongi* B (16)	KC684405	KC684343/KC684360/KC684377
*Polytremis jigongi*	130101066	male	West Tianmu Mountains, Zhejiang (WTMM)	30°20′N 119°23′E	26-Jul-09	*P. jigongi* B (16)	KC684405	KC684343/KC684360/KC684377
*Polytremis kiraizana*	121124051	male	Qilai, Taiwan (QL)	24°01′N 121°22′E	11-Jul-91	*P. kiraizana* (11)	KC684401	KC684340/KC684357/KC684374
*Polytremis suprema*	121124052	male	Jinxiu, Guangxi (JX)	24°07′N 110°11′E	31-Jul-11	*P. suprema* (13)	KC684402	KC684341/KC684358/KC684375
*Polytremis gotama*	130101067	male	Lijiang, Yunnan (LJ)	26°51′N 100°13′E	28-Jul-06	*P. gotama* (8)	KC684396	KC684351/KC684368/KC684385
*Borbo cinnara*	121124069	female	Taidong, Taiwan (TD)	22°59′N 120°59′E	17-Sep-07	*B. cinnara* (28)	KC684418	KC684354/KC684371/KC684388
*Pseudoborbo bevani*	121124067	male	Nantou, Taiwan (NT)	23°55′N 120°41′E	14-Sep-03	*P. bevani* (29)	KC684419	KC684353/KC684370/KC684387
*Pelopidas mathias*	–	–	–		–	*P. mathias* (30)	HQ990355*	–
*Pelopidas agna*	–	–	–		–	*P. agna* (31)	AB192485*	–
*Pelopidas Jansonis*	–	–	–		–	*P. Jansonis* (32)	AB192487*	–
*Parnara batta*	–	–	–		–	*P. batta* (33)	GU290263*	–
*Parnara guttata*	–	–	–		–	*P. guttata* (34)	HQ990728*	–
*Parnara ganga*	–	–	–		–	*P. ganga* (35)	GU290267*	–
*Parnara ogasawarensis*	–	–	–		–	*P. ogasawarensis* (36)	AB192490*	–
*Parnara bada*	–	–	–		–	*P. bada* (37)	GU290271*	–
*Polygonia caureum*	–	–	–		–	*P. caureum* (38)	JX445947*	KC413868*
*Lycaena phlaeas*	–	–	–		–	*L. phlaeas* (39)	AY556971*	KC413877*
*Cupido argiades*	–	–	–		–	*C. argiades* (40)	HQ004318*	KC413884*
*Pieris rapae*	–	–	–		–	*P. rapae* (41)	JQ996397*	KC413874*
*Pieris melete*	–	–	–		–	*P. melete* (42)	KC510141*	KC413844*
*Minois dryas*	–	–	–		–	*M. dryas* (43)	AB192478*	KC413854*
*Sericinus montela*	–	–	–		–	*S. montela* (44)	JX445945*	KC413855*

indicates the sequences retrieved from GenBank.

The nuclear DNA (D3+V4+V7) aligned data set contains 1048 nucleotide positions without gaps or stop codons, of which 42 positions are variable and 25 are parsimony informative. The mean base composition of the fragments shows no significant BIAS (T 24.3%, C 23.2%, A 23.8% and G 28.7%) (accession numbers KC684338–KC684388 and KC692371; Table 1). The average inter-specific K2P distance is 1.0%. The K2P distance between *P. theca theca* and *P. theca fukia* is 0.3%. *P. gotama* and *P. caerulescens* Mabille, 1876 share the same sequence.

### (b) Network of Genus *Polytremis*

**Figure 2:**
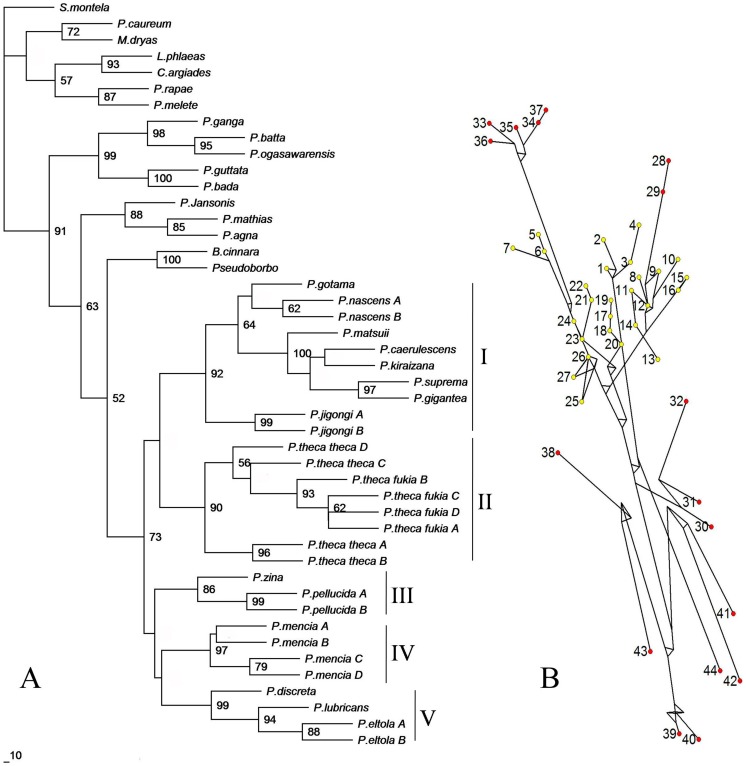
The ML tree and networks for COI. (A) Maximum-likelihood phylogeny on the basis of the mitochondrial COI sequences. A 50% majority-rule consensus bootstrap tree is shown, with bootstrap values over 50% at the nodes. (B) Network on the basis of the mitochondrial COI sequences constructed with software NETWORK4.5. In the figures, yellow dots indicate the haplotypes from Genus *Polytremis* and red dots indicate the haplotypes from outgroups. The sequences are labelled according to the numbers shown in Table 1.

Twenty-seven mitochondrial COI haplotypes of the genus *Polytremis* were used for network construction with the software Network4.5 using the median-joining method [17] along with sequences of other butterflies retrieved from GenBank (presented in Table 1). The results of network are shown in Fig. 2B. The network of concatenated sequences (COI+rDNA) gives substantially the same network of relationships as that of COI (data not shown).

### (c) Phylogenetics

**Figure 3:**
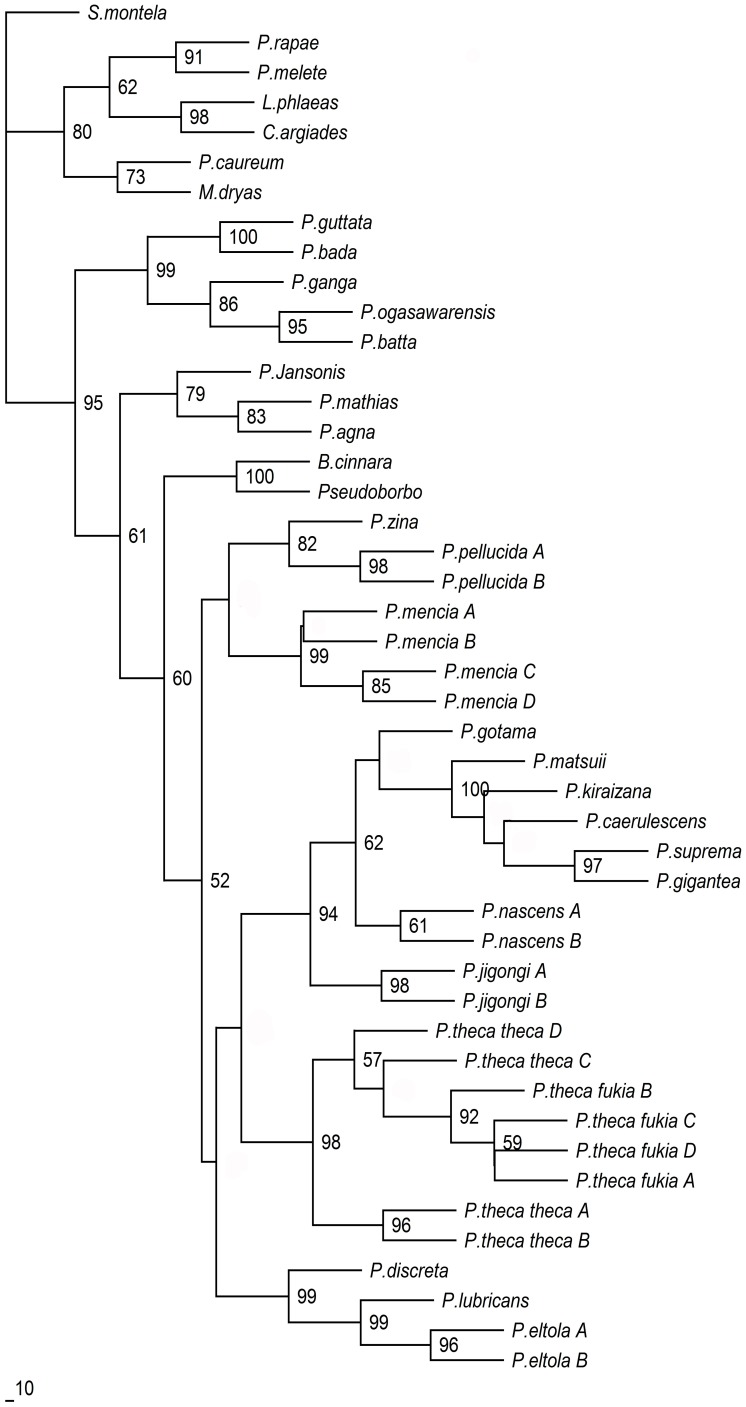
Maximum-likelihood phylogeny on the basis of the concatenated sequences (mitochondrial COI+nuclear rDNA) haplotypes. A 50% majority-rule consensus bootstrap tree is shown.

Figures 2A shows the ML tree based on the data set of COI and reveals five main clades. Clade I contained 8 species: *P. caerulescens*, *P. kiraizana* Sonan, 1938, *P. matsuii* Sugiyama, 1999, *P. suprema* and *P. gigantea* are first clustered with a strong support value. This is followed by clustering of *P. gotama* and *P. nascens* Leech, 1893. Finally, the newly described species *P. jigongi* Zhu, 2012 is revealed [4]. Two haplotypes of *P. jigongi* formstrongly supported lineages and are clearly separate from the other species. The genetic data confirms the validity of the new species. Clade II contains only *P. theca* reported to include three subspecies [5], [7], which shows a greater intra-specific genetic distance than some inter-specific genetic distances in the genus *Polytremis* (see above). Clade IV contains only one species, i.e., *P. mencia* Moore, 1877. Furthermore, the sister group relationship between *P. pellucida* Murray, 1875 and *P. zina* Evans, 1932**in Clade III is confirmed. Clade V contains three species, *P. lubricans* Herrich-Schäffer, 1869, *P. eltola* Hewitson, 1869 and *P. discreta* Elwes & Edwards, 1897, which are distributed sympatrically in the oriental region throughout India and Malaya. The ML tree of concatenated sequences (COI+ rDNA) gave substantially the same topologies as that of COI (Fig. 3).

### (d) Combined Morphological and Molecular Analysis

**Figure 4:**
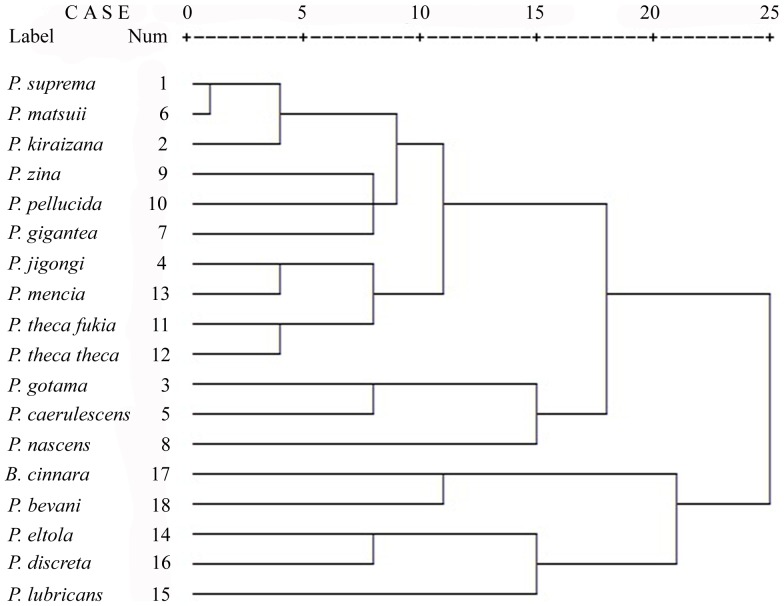
Dendogram corresponding to the Hierarchical cluster analysis using average between group linkage.

**Figure 5:**
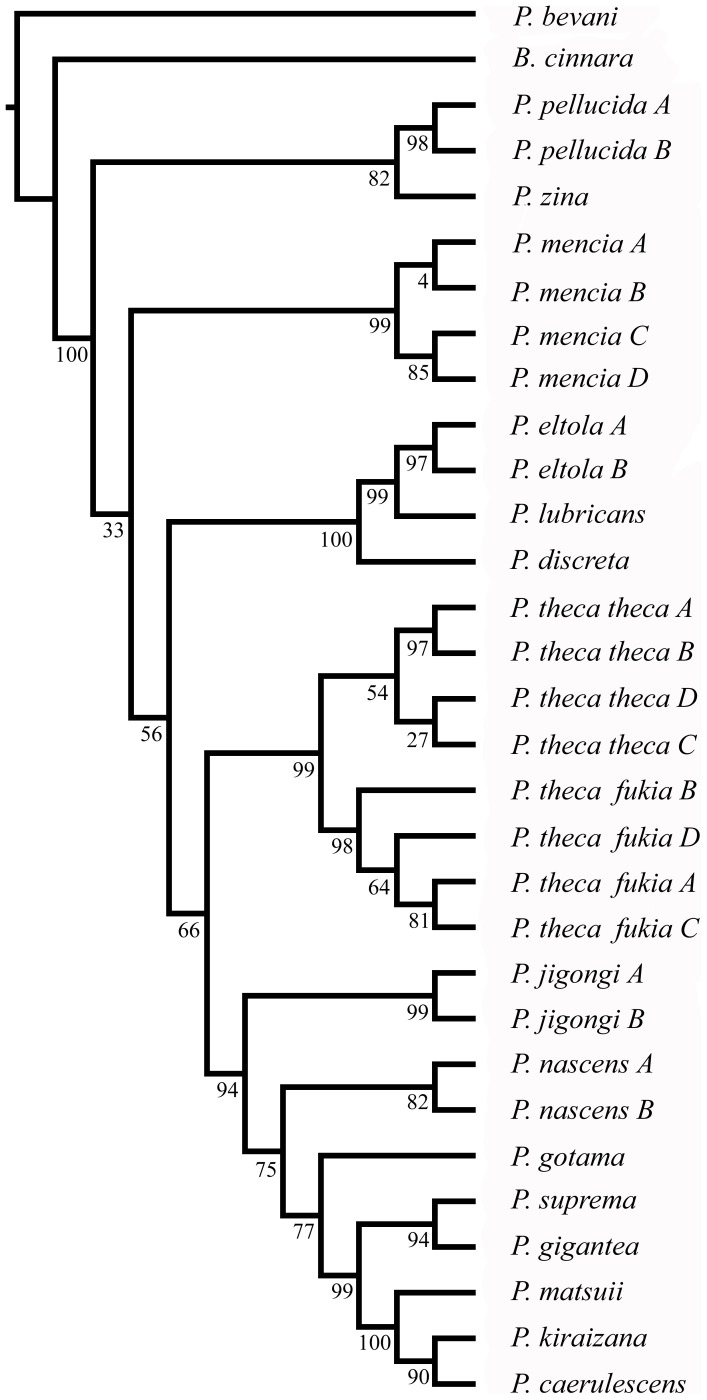
Support tree for the relationships within the genus *Polytremis* based on a combined analysis. Support tree based on 20 morphological characters and 4 genes. Supports for nodes are given in bootstrap values.

Hierarchical Cluster analysis of the 20 morphological characters yields a two-cluster solution (Fig. 4). The first cluster includes 12 species of *Polytremis*. The second includes the remaining three species and the outgroups. The combined analysis of morphological and molecular data returns three equally parsimonious trees with length (*L*) 328 steps, consistency index (HI) 0.6799 and retention index (RI) 0.8323. All supported clades from the combined data matrix are clades also appearing when the molecular data matrix is analysed on its own (Fig. 5).

## Discussion

### (a) Genetic Divergence

The level of DNA sequence divergence reflected the taxonomic hierarchy of the original species. On the basis of our data, the lowest intra-specific COI genetic distance was observed between *P. gigantea* and *P. suprema* (K2P distance 1.7%). Intra-specific distances, with only one exception (*P. theca*), were shorter. The COI data confirmed the sister group relationship between *P. gigantea* and *P. suprema*, which form a monophyletic group together with *P. caerulescens*, *P. kiraizana* and *P. matsuii*. All of their inter-specific distances were <3% (K2P distance). This was not surprising, because the five species shared many morphological traits inclding absence of a cornuti, thin coecum penis and ear-like uncus with a pair of processes.

We found that *P. theca* was the only species for which the intra-specific genetic distance was greater than some inter-specific genetic distances based on COI in the genus *Polytremis*. The distance was still much less than the average inter-specific genetic distances (K2P distance 7.9%) of the genus *Polytremis*. The species was widely distributed in the south of the Qinling Mountains, except in the Hainan Province and the southern tropical regions of Yunnan Province in China [5], [7]. A few subspecies were reported on the basis of morphological features of the wings [7]. Our specimens included two of them, namely *P. theca theca* and *P. theca fukia*. The COI tree revealed two distinct haplotype lineages without intermediates and the K2P distance was 4.2%. Additionally, the subspecies were separated by nuclear rDNA sequence (K2P distance 0.3%), suggesting the possible existence of a sibling species paired with allopatric distribution. This aspect warrants further study.

The average inter-specific rDNA genetic distance was 1.0% and far less than that of COI (K2P distance 7.9%). Except for *P. gotama* and *P. caerulescens*, other species in *Polytremis* could be distinguished with rDNA. *P. caerulescens* and *P. gotama* could be separated in the COI (K2P distance 1.9%), but showed no variation in the rDNA. The differences observed between results based on mitochondrial and nuclear markers may contribute to incomplete lineage sorting, introgressive hybridization or recent separation [18], [19]. Considering that *Polytremis* is quite an old genus and the splits of COI of the two lineages are also fairly old, it seems that incomplete lineage sorting may not be an appealing explanation for the discordance [20]. Additionally, *P. gotama* has been described as an independent species by morphological features [21] and our COI data of the three specimens of *P. caerulescens* (from two populations) and one specimen of *P. gotama* revealed two distinct haplotype lines with K2P distances of 1.9%, without intermediates, This observation may indicate that they were two species based on the morphological and molecular level. Nevertheless, more specimens from different population of the two species need to be collected and analyzed in future to see if this pattern is recovered consistently and further confirm the relationship of them. Instead, several arguments favor the recent separation assumption. As far as their morphological characteristics were concerned, *P. caerulescens* was considered to be closely related to *P. gotama* on the basis of the structural similarity of male genitalia and the cell spot on the upperside of the hindwing, which were not found in the other *Polytremis* species [22]. Additionally, only these two species were observed and captured at altitudes>2000 m (Zhu et al. unpublished data). *P. caerulescens* was a little larger than *P. gotama*. They both varied in other morphological traits, including the ground color of the wings and male stigma on the upperside of the forewing (see Table 2), which clearly support the existence of two closely related but distinct species. K2P distances of the COI fragments reached 1.9%, whereas rDNA showed no sequence variation, suggesting a possible recent separation of these two species.

**Table 2:** List of the morphological characters of Polytremis spp.

species	male stigma	harpe terminally	cell spot(s) on upperside of forewing	underside ground color	shape of a pair of processes on the dorsal portion of uncus	shape of phallus distally	coecum penis	length ratio of phallus and valva	spots on hind- wing in spaces M1 and M2	shape of fore- wing spot in space Cu1
*P. suprema*	1	0	1	1	0	1	0	0	1	0
*P. kiraizana*	1	1	1	1	0	1	0	0	1	0
*P. gotama*	1	0	0	1	0	1	0	0	1	0
*P. jigongi*	1	0	1	0	0	1	0	0	1	0
*P. caerulescens*	0	0	0	1	0	1	0	0	1	0
*P. matsuii*	1	0	1	1	0	1	0	0	1	0
*P. gigantea*	0	0	1	1	0	1	0	1	1	0
*P. nascens*	1	1	0	1	0	1	0	0	1	0
*P. zina*	0	0	1	1	0	1	1	0	1	0
*P. pellucida*	0	0	1	1	-	0	0	0	1	0
*P. theca fukia*	0	0	1	0	0	1	0	0	1	0
*P. theca theca*	0	0	1	1	0	1	0	0	1	0
*P. mencia*	1	1	1	0	0	1	0	0	1	0
*P. eltola*	0	0	1	1	0	1	1	0	0	0
*P. lubricans*	0	0	1	1	1	1	0	0	1	1
*P. discreta*	0	0	1	1	0	1	0	1	0	0
*B. cinnara*	0	0	1	0	-	1	1	0	1	0
*P. bevani*	0	0	1	1	0	1	1	0	1	0

species	color of spots on underside of hindwing	a silver short line in discal cell on underside of hindwing	cornuti	spots on both wings	color of semi- hyaline spots on forewing	spots on upperside of hindwing	Coecum penis	Prominence in the back of Tegumen	Bifid uncus	Sharp elongated harpe
*P. suprema*	0	0	0	1	0	1	0	0	1	1
*P. kiraizana*	0	0	0	1	0	1	0	0	1	1
*P. gotama*	0	1	0	0	0	0	0	0	1	1
*P. jigongi*	0	0	1	1	0	1	0	0	1	1
*P. caerulescens*	0	0	0	0	0	0	0	0	1	1
*P. matsuii*	0	0	0	1	0	1	0	0	1	1
*P. gigantea*	0	0	0	1	0	1	0	0	1	1
*P. nascens*	0	0	1	0	0	1	0	0	1	1
*P. zina*	0	0	0	1	0	1	0	0	1	1
*P. pellucida*	0	0	0	1	0	1	0	0	1	1
*P. theca fukia*	0	0	1	1	0	1	0	0	1	1
*P. theca theca*	0	0	1	1	0	1	0	0	1	1
*P. mencia*	0	0	1	1	0	1	0	0	1	1
*P. eltola*	1	0	0	1	1	1	0	0	1	1
*P. lubricans*	1	0	0	1	1	1	0	0	1	1
*P. discreta*	1	0	0	1	1	1	0	0	1	1
*B. cinnara*	1	0	0	1	1	1	1	1	1	0
*P. bevani*	1	0	0	1	1	1	0	0	0	0

### (b) DNA Barcoding and Tree-based Identificaion

Ideally, inter-specific divergence should be about 10 times higher than intra-specific divergence [23], which could form a barcoding gap between inter- and intra-species. However, the absence of a gap in some studies led researchers to caution against the use of a simple distance threshold-oriented barcoding approach [24]–[26]. As far as the studies of Lepidoptera are concerned, Hajibabaei et al [27] found such a gap in their dataset while Wiemers and Fiedler [26] failed to confirm this finding in their studies. Meyer and Paulay [24] assume that insufficient sampling on both the inter-specific and intra-specific level are responsible for the barcoding gap, while others argue that the main reason for an overlap can be found in inappropriate assumptions underlying a sequence from the DNA library (i.e. poor identification, alpha-taxonomy or incompatible species criteria).

**Figure 6:**
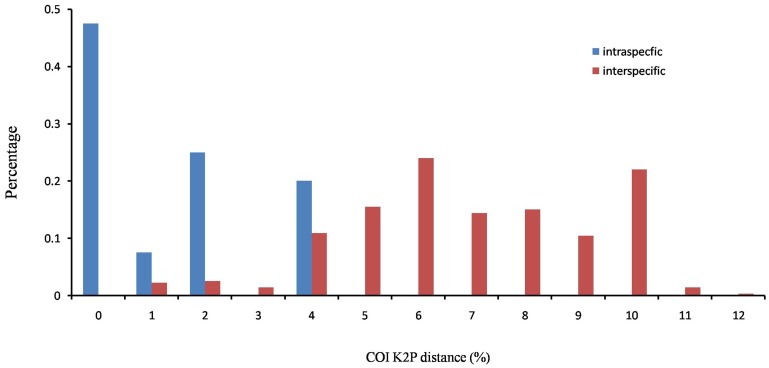
Distribution of the intra-specific and inter-specific genetic variabilities (K2P distance) of cytochrome c oxidase I.

Although thresholds have been proposed in Lepidoptera as 3% for COI [28], intra- and inter-specific genetic divergences did not fall into separate intervals and an obvious ‘barcode gap’ did not occur in COI in our study of *Polytremis* (Fig. 6). It was entailed by two factors. Firstly, The Interspecific K2P distances among five sister species (*P. gigantean*, *P. suprema*, *P. caerulescens*, *P. kiraizana* and *P. matsuii*) were less than 3%, which caused the intra- and inter-specific genetic divergences overlap from 1.7% to 3%. Secondly, all intra-specific distances were less than 3% except for *P. theca*. However, we inferred the subspecies of *P. theca* could be a sibling species pair according the molecular and morphology data in the study. Regardless, for COI, the overlaps between intra- and inter-specific variations would not affect identification in a thoroughly sampled evaluation [24]. The *Polytremis* species could be distinguished by tree-based methods and clustered with a well support, suggesting the COI sequence could be used as a DNA barcode to correctly identify almost all species in the genus (Fig. 2A).

The markers of the nuclear rDNA sequences used in our studies have been proposed as a reasonable alternative to mitochondrial COI. These markers could identify or delimit species or species-like units. As they are not inherited maternally and avoid problems of mitochondrial markers such as introgression and pseudogenes [29].

### (c) Analyses of Molecular and Morphological Data

ML tree constructed in this study based on COI showed that specimens belonging to the same species formed a monophyletic cluster including the new species, *P. jigongi*, whose earlier descriptions were based on morphology and geographical distribution [4]. Meanwhile, there was considerable congruence in topology of the inter-species level for both mtDNA COI and concatenated sequences ML trees indicating certain clades were well differentiated phylogenetically (Fig. 2, 3). The strong support for the monophyly of *Polytremis* was found in the analyses of the COI and concatenated alignments.

The results obtained by Hierarchical Cluster analysis showed traditional classification were basically consistent with molecular phylogeny of the genus *Polytremis* (Fig. 4). However, the morphological analysis resulted in only limited resolution based on just 20 morphological characters since the morphological characters and character states were commonly homologous in *Polytremis*. On contrary, molecular classification provided a more precise, lower artificial taxonomic rank. Thus, the combination of the morphological and molecular matrix was better resolved for understanding of the phylogeny of this genus.

## Materials and Methods

### (a) Ethics Statement

No specific permits were required for the described studies. No specific permissions were required for these locations. The location from where we collected the insects is not privately-owned or protected in any way. The insects used in the studies did not involve endangered or protected species. During the experiment, the insects were never maltreated.

### (b) Samples Collection

**Figure 7:**
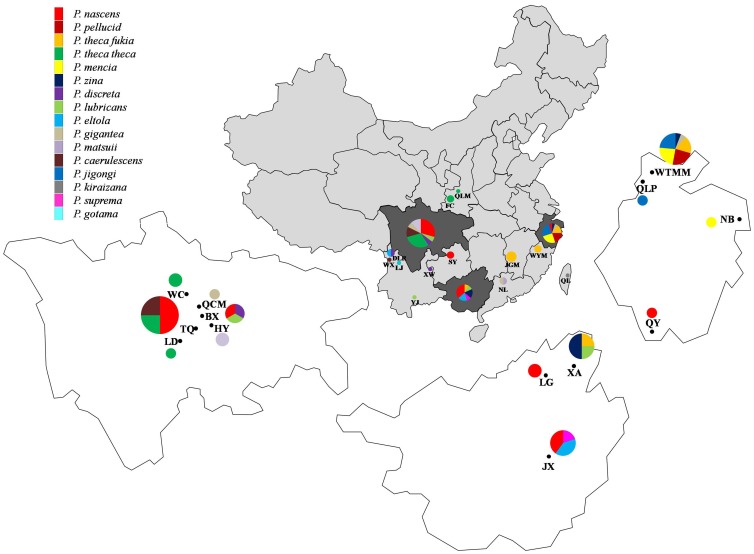
Distribution of the specimens of genus *Polytremis* collected in China. For full site names and other details see Table 1.

We collected 67 specimens from 15 of the estimated 18 species in the genus *Polytremis*, including the newly reported *P. jigongi* [4], from different localities (Fig. 7, Table 1). All specimens were caught in the field and preserved by dehydration in small envelopes. The preliminary species-level identification was based on traditional morphological characteristics of wings, genitalia (see Table S1), locality and additional information [22]. *Borbo cinnara* and *Pseudoborbo bevani*, two species classed in the same tribe as *Polytremis* spp. served as outgroups in the phylogenetic analyses.

### (c) Molecular Methods

The DNA was isolated from leg tissue using a QIAamp DNA Mini kit (QIAGEN, Hilden, Germany) essentially following the manufacturer’s instructions but with some modification. Briefly, after adding proteinase K and buffer AL (QIAGEN®), the mixed homogeneous solution was incubated at 70°C for 2 h. Subsequently, 200 µL of 100% ethanol was added and the mixture transferred to a QIAamp spin column. The mixture in the spin column was subjected to 3 cycles of centrifugation at full speed (14,000 *g*) for 1 min and the filtrate was returned to the spin column to increase the amount of DNA obtained.

We amplified approximately 490 bp of the mitochondrial COI gene, recommended as the universal and standard barcoding marker for species identification [23], [30], using the primers RON (5'- GGA TCA CCT GAT ATA GCA TTC CC -3') and NANCY (5'- CCC GGT AAA ATT AAA ATA TAA ACT TC -3') [31]. Amplification of the targeted regions by PCR was done in 20 µL volumes with 50–100 ng template DNA (i.e. with DNA concentrations of 2.5–5 ng/µL), 0.4 µM each of the amplification primers, 200 µM each dNTP, 1.5 mM MgCl_2_, 50 mM KCl, 10 mM Tris–HCl (pH8.3) and 2 U Taq polymerase (Takara, Otsu, Shiga, Japan). The PCRs were run on a DNA thermal cycler (Bio-Rad, Hercules, CA, USA) with cycling parameters: 95°C for 5 min followed by 35 cycles of 94°C for 45 s, 42°C for 1 min, 90°C for 90 s and a final elongation step at 72°C for 10 min.

For nuclear DNA, We selected three expansion segments (known as variable domains) of 18S rDNA and 28S rDNA, the slowly evolving genes used normally in higher classification studies [32], [33]. The faster evolving mitochondrial gene and highly conserved nuclear genes were included in order to resolve deep polyphyletic structuring inferred in the earlier study by Fan [34]. The author divided the genus into two genera, *Polytremis* and *Zinaida* Evans, 1939, on the basis of the morphological and molecular data with a limited sample of 7 species. The ∼240 bp long D3 region was amplified with primers CD3F and CD3R (5'- GGA CCC GTC TTG AAA CAC -3' and 5'- GCA TAG TTC ACC ATC TTT C -3') using a PCR protocol of 94°C for 5 min, then 32 cycles at 94°C for 45 s, annealing at 52°C for 45 s, extension at 72°C for 80 s and a final extension step at 72°C for 7 min. The ∼390 bp region of the V4 gene fragment was amplified with the primer pair CV4F (5'- TGG TGC CAG CAG CCG CGG TAA -3') and CV4R (5'- CCT CTA ACG TCG CAA TAC GAA TGC CC -3'). The PCR temperature protocol was: 94°C for 5 min then 32 cycles of denaturation at 94°C for 45 s, annealing at 66°C for 45 s, extension at 72°C for 2 min and a final extension step at 72°C for 8 min. Finally, an ∼400 bp long region of the V7 gene fragment was amplified using the forward primer CV7F (5'- CTT AAA GGA ATT GAC GGA GGG CAC CAC C -3') and the reverse primer CV7R (5'- GAT TCC TTC AGT GTA GCG CGC GTG -3') with the following PCR conditions: 94°C for 5 min then 32 cycles of denaturation at 94°C for 45 s, annealing at 68°C for 45 s, extension at 72°C for 2 min and a final extension step at 72°C for 8 min [19]. Extraction blanks were run in all reactions to control for contamination during the extraction and PCR processes. The amplification products were subjected to electrophoresis in a 2% (w/v) agarose gel in TAE buffer (0.04 M Tris–acetate, 0.001 M EDTA) with a DL1000 ladder size marker (Takara, Otsu, Shiga, Japan) to determine whether the amplification reactions were successful. In addition, after electrophoresis in agarose gels, the amplification products from leg tissues were extracted using the Wizard SV Gel and PCR Clean-up System (Promega, Madison, WI, USA) for sequencing. Finally, all the sequences obtained were deposited in GenBank (Table 1).

### (d) Molecular Analysis

The sequence data of the mitochondrial COI, nuclear rDNA and concatenated sequences (mitochondrial COI+nuclear rDNA) were aligned with published homologous sequence from butterflies in the same tribe as *Polytremis* spp., (e. g., *Parnara* spp., *Pelopidas* spp. etc.) as well as some out of the tribe (presented in Table 1), translated to amino acid sequences to check for nuclear mitochondrial pseudogenes (numts) and pruned to remove redundant sequences with Bioedit v.7.0 [35]. The haplotype sequence matrix was used for all subsequent phylogenetic analyses (Table 1). For the COI and the concatenated data set, the substitution saturation was determined with DAMBE v.4.5 by plotting numbers of transitions and transversions against the Kimura 2-parameter distance (K2P) [36], [37]. MEGA v4.0 was used to calculate the intra- and inter-specific genetic divergences based on the K2P model [38]. Phylogenetic trees were constructed by the ML methods using PAUP 4.0b10 [39]. Relationships among the mitochondrial COI and concatenated sequences (mitochondrial COI+nuclear rDNA) haplotypes were represented as a haplotype network obtained by the software DnaSP4.90 [40] and Network4.5 (fluxus-engineering.com) using the median-joining method [17].

### (e) Analysis of Some Morphological Features

To investigate the morphological variation between *Polytremis* species, we dissected, photographed and described a total of 20 morphological features of genitals and wings. These features recommended by Evans [5], Eliot [41] and Chou [42] were used to recognize groups and/or species within *Polytremis*. The description of morphological characters in each species is given in Table 2. A rectangular raw data matrix was constructed that contains 18 rows (species) and 20 columns (morphological variables). The Hierarchical Cluster procedure of the SPSS package (version 11.5, Chicago, IL, 2001) was employed using squared Euclidean distance as the measure and between-groups linkage as the cluster method. Twenty morphological variables are:

1. Male stigma: absent (0), present (1)
2. Harpe terminally: integrity (0), bifid (1)
3. Cell spot(s) on upperside of forewing: single cell spot (0), two cell spots (1)
4. Underside ground color: greenish ochreous (0), brown (1)
5. Shape of a pair of processes on dorsal portion of uncus: ear-like (0), hook-like (1)
6. Shape of phallus distally: integrity (0), bifid (1)
7. Coecum penis: thin (0), thick (1)
8. Length ratio of phallus and vulva: <2 (0), ≥2 (1)
9. Spots on hindwing in spaces M1 and M2: conjoined (0), separate (1)
10. Shape of forewing spot in space Cu1: normal (0), elongate (1)
11. Color of spots on underside of hindwing: white (0), yellowish (1)
12. A silver short line in discal cell on underside of hindwing: absent (0), present (1)
13. Cornuti: absent (0), present (1)
14. Spots on both wings: inconspicuous (0), conspicuous (1)
15. Color of semi-hyaline spots on forewing: white (0), yellowish (1)
16. Spots on upperside of hindwing: absent (0), present (1)
17. Coecum penis: normal (0), expansion to both sides
18. Prominence in the back of Tegumen: absent (0), present (1)
19. Bifid uncus: absent (0), present (1)
20. Sharp elongated harpe: absent (0), present (1)

### (f) Combined Analysis of Morphological and Molecular Data

The various data partitions (morphological and molecular data) originated as Nexus files, opened with the program Mesquite [43], exported in Hennig86 /Nona format, and were opened and combined with the program Winclada [44]. Data were exported from Winclada and analyzed in TNT [45]. All analyses employed all four new technology search methods [46], [47], using the default settings in TNT, except: the ratchet weighting was probability 5% and there were 200 iterations; tree drifting used 50 cycles; tree-fusing used five rounds; minimum length was set to be hit 25 times. Molecular gaps were treated as missing data. Stability of the nodes of the most parsimonious trees was assessed using 1000 bootstrap pseudoreplicats.

## Supporting Information

Table S1 **Key to the species of genus** ***Polytremis.***
(DOCX)
