# Supplementary material for: Molecular Phylogeny of the Butterfly Genus Polytremis (Hesperiidae, Hesperiinae, Baorini) in China
Source: PLoS One. 2013 Dec 31;8(12):e84098. doi: 10.1371/journal.pone.0084098 (PMC3877198; doi:10.1371/journal.pone.0084098)
Supplement: Table S1 — Key to the species of genus Polytremis. (DOCX) [file pone.0084098.s001.docx]

**Table S1—— Key to the species of genus *Polytremis***

1. Forewing upperside with lower cell spot…………………………………………………………………………………………….. 2

Forewing upperside without lower cell spot …………………………………………………………………………….……….….15

1. Forewing upperside with upper cell spot …………………………………………………………………………….……………….3

Forewing upperside without upper cell spot, very small in size ………………………………………………….…………*P. minuta*

1. Forewing cell spots conjoined………………………………………………………………………………………………………...4

Forewing cell spots separated…………………………………………………………………………………………………………6

1. Forewing upperside with a spot in space Cu2, hindwing spots in M1 and M2 conjoined …………………………………………...5

Forewing upperside without a spot in space Cu2, hindwing spots in M1 and M2 separated; forewing apex white……….*P. annama*

1. Phallus long, over twice length as the valva; harpe thick …………………………………………………………………..*P. discrete*

Phallus short, about 1.5 times length as the valva; harpe slender ……………………………………………………………. *P. eltola*

1. Forewing lower cell spot elongated……………………………………………………………………………………………………7

Forewing lower cell spot not elongated………………………………………………………………………………………………11

1. Forewing upperside with a stigma in space Cu2 ………………………………………………………………………………………8

Forewing upperside without a stigma in space Cu2…………………………………………………………………………………..10

1. End of phallus with two processes, not equal in length………………………………………………………………………*P. matsuii*

End of phallus with two processes, equal in length…………………………………………………………………………………….9

1. Harpe terminally bifid……………………………………………………………………………………………………….*P. kiraizana*

Harpe terminally not bifid, integrity and pointed…………………………………………………………………………….*P. suprema*

1. Phallus deeply bifid distally, harpe sharply pointed terminally……………………………………………………………..*P. gigantean*

Phallus slightly bifid distally, harpe blunt terminally……………………………………………………………………..……...*P. zina*

1. Spots yellow in colour, forewing spot in space Cu1 elongated………………………………………………………..……*P. lubricans*

Spots white in colour, forewing spot in space Cu1 never elongated………………………………………………………..…………12

1. Uncus upperside with a pair of processes ………………………………………………………………………………..……………13

Uncus upperside without a pair of processes……………………………………………………………………….…….….*P. pellucida*

1. Forewing upperside with a stigma in space Cu2………………………………………………………………….……………………14

Forewing upperside without stigma, harpe shot and pointed at tip……….………….………….………….…...…….…………*P. theca*

1. Stigma linear shaped; harpe distally with a process……….………….………….………….………….………….………….,*P. mencia*

Stigma often broken; harpe distally without process……….………….………….………….………….………….………….*P. jigongi*

1. Hindwing underside spots pale purple in colour……….………….………….………….………….………….………..*P. caerulescens*

Hindwing underside spots white in colour……….………….………….………….………….………….………….………….……..16

1. Phallus with cornuti, spots in forewing obvious ……….………….………….………….………….………….………….………….17

Phallus without cornuti, spots in forewing small and obscure……….………….………….………….………….…………..*P. gotama*

1. Stigma always broken in two, harpe with a process outwardly……….………….………….………….………….…………*P. nascens*

Stigma linear shaped, harpe integrity, without process outwardly……….………….………….………….…………..*P. micropunctata*
